# ADME and Pharmacokinetic Properties of Remdesivir: Its Drug Interaction Potential

**DOI:** 10.3390/ph14070655

**Published:** 2021-07-08

**Authors:** Subrata Deb, Anthony Allen Reeves, Robert Hopefl, Rebecca Bejusca

**Affiliations:** Department of Pharmaceutical Sciences, College of Pharmacy, Larkin University, Miami, FL 33169, USA; aallen@ularkin.org (A.A.R.); rhopeful@myularkin.org (R.H.); rbejusca@myularkin.org (R.B.)

**Keywords:** remdesivir, ADME, pharmacokinetics, COVID-19, cytochrome P450, drug interaction, transporter

## Abstract

On 11 March 2020, the World Health Organization (WHO) classified the Coronavirus Disease 2019 (COVID-19) as a global pandemic, which tested healthcare systems, administrations, and treatment ingenuity across the world. COVID-19 is caused by the novel beta coronavirus Severe Acute Respiratory Syndrome Coronavirus 2 (SARS-CoV-2). Since the inception of the pandemic, treatment options have been either limited or ineffective. Remdesivir, a drug originally designed to be used for Ebola virus, has antiviral activity against SARS-CoV-2 and has been included in the COVID-19 treatment regimens. Remdesivir is an adenosine nucleotide analog prodrug that is metabolically activated to a nucleoside triphosphate metabolite (GS-443902). The active nucleoside triphosphate metabolite is incorporated into the SARS-CoV-2 RNA viral chains, preventing its replication. The lack of reported drug development and characterization studies with remdesivir in public domain has created a void where information on the absorption, distribution, metabolism, elimination (ADME) properties, pharmacokinetics (PK), or drug-drug interaction (DDI) is limited. By understanding these properties, clinicians can prevent subtherapeutic and supratherapeutic levels of remdesivir and thus avoid further complications in COVID-19 patients. Remdesivir is metabolized by both cytochrome P450 (CYP) and non-CYP enzymes such as carboxylesterases. In this narrative review, we have evaluated the currently available ADME, PK, and DDI information about remdesivir and have discussed the potential of DDIs between remdesivir and different COVID-19 drug regimens and agents used for comorbidities. Considering the nascent status of remdesivir in the therapeutic domain, extensive future work is needed to formulate safer COVID-19 treatment guidelines involving this medication.

## 1. Introduction

Coronavirus Disease 2019 (COVID-19) is arguably the single most serious health concern that has impacted the entire world in modern times within such a short period of time. According to the World Health Organization (WHO), as of 21 May 2021, there have been 165,069,258 cases worldwide, resulting in 3,422,907 deaths [1]. COVID-19 is a disease caused by the novel beta coronavirus known as Severe Acute Respiratory Syndrome Coronavirus 2 (SARS-CoV-2). It is commonly accepted that the entire coronavirus family is zoonotic, and SARS-CoV-2 can be specifically traced back to native populations of bat [2]. The first reported case of COVID-19 was in December 2019, from the city of Wuhan (Hubei Province, China) where it was originally coined as pneumonia of unknown origin [3]. COVID-19 was officially classified as a pandemic by the WHO on 11 March 2020 [4].

SARS-CoV-2 is an incredibly infectious genetic variant of past coronaviruses (MERS-CoV, SARS-CoV), which belong to the family *Coronavirdae*. This family of viruses are single stranded RNA viruses and received their name for the spike-like projections on their surface (*Corona*, translating to crown in Latin) [5]. Of the four subgroups (alpha, beta, gamma, delta) within this family, the beta viruses are the most infectious, and is the lineage to the very pathogenic SARS-CoV and MERS-CoV [6]. SARS-CoV-2 varies from other coronaviruses via mutations D614 and G614 occurring in the spike protein of the virus. It has been hypothesized that the G614 mutation may result in higher transmission rates than D614 [7]. Transmission of SARS-CoV-2 has been determined to be through either respiratory droplets or fecal contamination and can occur via human to human, animal to human, or encountering infected surfaces [8].

Since the beginning of the pandemic, several investigational drug options have been explored with limited success. Various health agencies across the globe have identified that social distancing, use of alcohol-based hand sanitizers, frequent hand washing with soap, and use of masks have been the most effective ways of preventing the transmission of the infection [4,9]. However, depending on the severity, patients infected with COVID-19 need a multitude of efforts to manage their symptoms. The range of approaches include interference with the entry of SARS-CoV-2 and viral genome replication cycle, lowering of inflammation, and management of oxygen levels [10,11]. The repurposing of the existing drugs led to the use of following agents as investigational treatment of COVID-19: agents inferring with the virus replication (remdesivir, chloroquine, hydroxychloroquine, lopinavir, ritonavir) and anti-inflammatory drugs (corticosteroids, interleukin-1 inhibitors, interleukin-6 inhibitors such as sarilumab, siltuximab, tocilizumab, interferons alfa-2b and beta-1a, and Janus kinase inhibitors such as baricitinib) [12,13]. Among these agents, remdesivir has been recently approved by US Food and Drug Agency (FDA) and European Medicines Agency (EMA) for use in COVID-19 patients [14,15]. However, due to the nascent nature of the drug, there is currently limited information available about the absorption, distribution, metabolism, and elimination (ADME) properties, the pharmacokinetics (PK), or the drug-drug interactions (DDI) of remdesivir. Carboxylesterase enzymes, cytochrome P450 (CYP) enzymes, and transporters are involved in the disposition of remdesivir, thus making the drug susceptible to drug-drug interactions with the agents used to manage COVID-19 symptoms and with the therapeutics for comorbidities. In this narrative review, we have evaluated the currently available information on the ADME, PK, and DDI of remdesivir, and have also predicted the potential DDIs involving remdesivir based on its metabolic profile and ability to interfere with drug metabolizing enzymes and transporters. The aim of the present work is to provide a summary of remdesivir disposition and offer a mechanistic framework to predict remdesivir-related drug interactions in COVID-19 patients.

## 2. Remdesivir: Chemical and Pharmacological Properties

Remdesivir has been one of the very few drugs that has shown limited success in the treatment of COVID-19 [2]. The drug was originally developed to treat Ebola virus but has been shown to have broad spectrum antiviral activity including against SARS-CoV-2 and MERS [16]. These characteristics have brought remdesivir to the forefront for treatment of SARS-CoV-2 and eventually led to first its emergency use authorization by US FDA on 1 May 2020, and subsequent FDA approval on 22 October 2020. Currently, remdesivir is indicated for hospitalized adult and pediatric COVID-19 patients in the age range of twelve years and older weighing ≥40 kg. In October 2020, the FDA also issued an emergency use authorization for very young patients with the weight of 3.5 kg to <40 kg or for patients younger than twelve years with a weight of no less than 3.5 kg [17,18].

**Figure 1:**
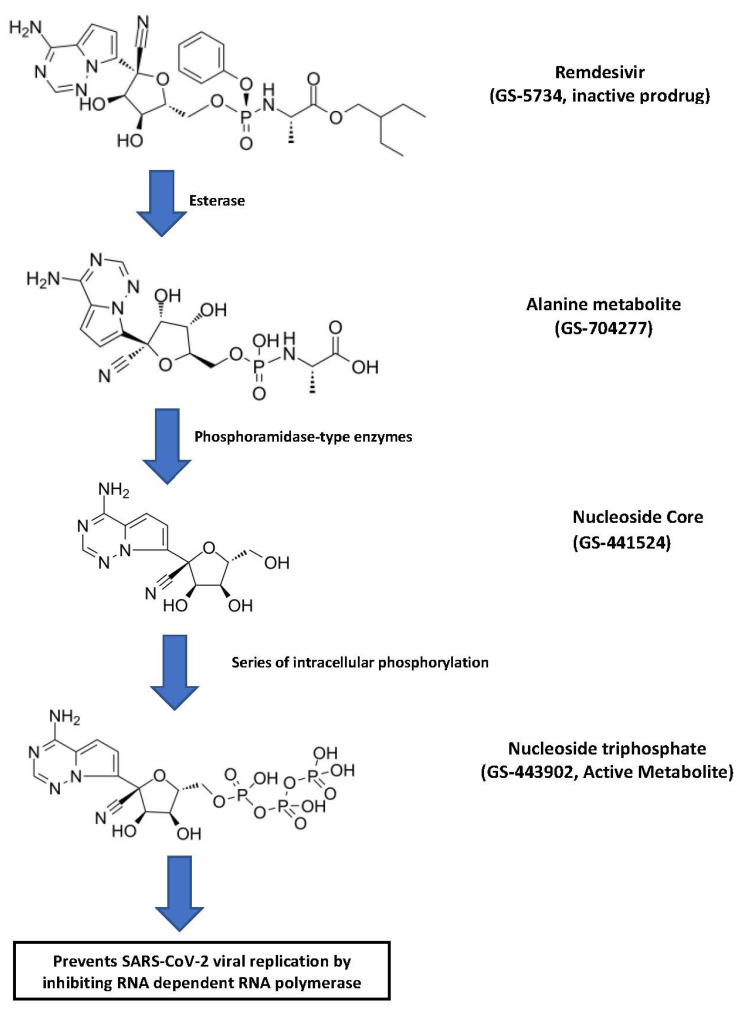
Step-wise bioactivation of remdesivir [15,18].

Remdesivir (GS-5734) is an adenosine analog prodrug that is converted first to the alanine metabolite (GS-704277) and then to the nucleoside core (GS-441524) (Figure 1).

Intracellularly, the nucleoside core is converted to nucleoside monophosphate and finally to pharmacologically active nucleoside triphosphate metabolite (GS-443902) [2]. The active remdesivir triphosphate competes with endogenous adenosine triphosphate for incorporation into SARS-CoV-2 RNA chains and blocks the SARS-CoV-2 RNA-dependent RNA polymerase enzyme. The incorporation of remdesivir triphosphate into viral RNA results in delayed chain termination during viral RNA replication [2]. The molecular formula of remdesivir is C_27_H_35_N_6_O_8_P, and the molecular weight is 602.6 g/mol. There are six chiral centers in the molecule, but the drug is manufactured as a single stereoisomer. There are various polymorphic physical forms, however, the active substance is in form II or a mixture of form II and another crystalline form. Both the mixture and the pure form show similar solubility and yield similar product performance [15].

Remdesivir is administered as a slow infusion (30–120 min) of a single loading dose (200 mg/day), followed by maintenance dose (100 mg/day) for a total of either five or ten days [18]. The incidences of grades ≥3 adverse reactions, serious adverse reactions, and suspension of drug administration were actually lower in the remdesivir-treated group compared to the placebo group. However, these categories of adverse effects were higher in 10-day treatment group compared to the 5-day treatment group [18]. Several biochemical and physiological abnormalities were observed including liver functions, renal function, increase in glucose and prothrombin time, and decreased hemoglobin and lymphocytes. Increase in transaminase activities (alanine transaminase and aspartate transaminase) and decreased creatinine clearance and estimated glomerular filtration rate (eGFR) prompted monitoring during and immediately after the treatment period [15]. It is important to recognize that the liver and kidney functions were more affected in the placebo group, likely due to the untreated COVID-19 pathophysiology, triggering those effects through inflammatory pathways [19].

## 3. ADME Properties of Remdesivir

### 3.1. Absorption

Remdesivir is available in two formulations: a solution and a lyophilized powder. The solution is formulated as 5 mg/mL in a vial of 100 mg/20 mL and the lyophilized powder is packaged as 100 mg in a single-dose vial. The powder formulation needs to be reconstituted with either a 0.9% saline or 5% glucose solution [20]. The most favorable route of administration is intravenous infusion over 30 min to 120 min. In adults, remdesivir is administered as follows: 200 mg intravenous loading dose on day one, followed by daily doses of 100 mg intravenously either for 2–5 days or up to 10 days [18]. Intravenous administration enables 100% absorption of remdesivir, which is why it is the preferred route. Oral administration is not indicated because it undergoes very high hydrolysis-mediated first-pass clearance in the gastrointestinal tract leading to limited absorption and systemic concentration [20,21]. Remdesivir has been formulated as a prodrug to increase the cellular concentration of remdesivir triphosphate [21]. Intramuscular administration, although better than oral, would result in subtherapeutic levels as well, since remdesivir slowly releases from the muscles, thus causing blood concentrations to be low and out of therapeutic range [21]. The inhaled route of administration of remdesivir is currently being explored since SARS-CoV-2 first targets the lungs and it is presumed that absorption would be adequate to deliver the drug right into the primary infected area. The inhaled formulation is mainly targeted for outpatient use and for less serious COVID-19 patients [21,22]. Following slow infusion, the remdesivir prodrug diffuses through the cellular membrane and undergoers a series of hydrolysis reactions in the cytosol [2]. The hydrolysis of nucleoside core (GS-441524) leads to a more water-soluble monophosphate form which is unable to travel back and hence remains in the infected cells [2]. Thus, the ester prodrug of remdesivir is efficiently absorbed once administered as a slow intravenous infusion. In addition to the dosage form and route of administration, the absorption of remdesivir can be influenced by transporters. Remdesivir is a substrate of P-glycoprotein (P-gp) and organic anion transporting polypeptides 1B1 (OATP1B1) transporters. Similarly, the alanine metabolite of remdesivir is also a substrate for OATP1B1 and OATP1B3 [15]. Depending on the transporter expression profile in the cell membrane, P-gp can efflux remdesivir out of the cellular compartment, leading to lower intracellular levels and effectiveness.

### 3.2. Distribution

After intravenous administration of remdesivir, the drug goes through passive diffusion to reach the tissues and blood cells [20]. The parent remdesivir has relatively high affinity for human plasma proteins with 88.0–93.6% binding, whereas GS-441524 and GS-704277 have 2% and 1% protein binding, respectively [20]. Remdesivir is expected to have low tissue distribution because of its instability within the tissues; however, once its active metabolite enters the cells it accumulates more than the extracellular prodrug form [15]. Indeed, in a cohort of eight subjects, remdesivir showed a volume of distribution of about 45.1–73.4 L with a single dose of 10 mg to 225 mg [23]. Similarly, a multiple-dose (150 mg/day) study of 7 days or 14 days demonstrated volume of distribution of 85.5 L. The concentrations of GS-441524 (nucleoside core) and GS-443902 (active triphosphate) in peripheral blood mononuclear cells (PBMCs) were used as a marker of distribution of remdesivir in cellular compartment. It is notable to say that the intracellular concentrations were 220 times to 370 times higher than the EC_50_ for SARS-CoV-2 [23]. There is some animal data in rats and monkeys that show a limited distribution of remdesivir to most tissues, but little or no evidence of remdesivir presence was found in the brain, indicating it does not cross the blood–brain barrier well [15]. This suggests the need for higher or more frequent dosing to achieve higher concentrations in the infected organs. However, remdesivir doses higher than 200 mg have been linked to increased hepatotoxicity and renal dysfunction [21]. Thus, there is a need for further development of an inhaled dosage form, so that remdesivir can be distributed in the optimum concentrations to the infected cells while decreasing systemic toxicity and adverse drug reactions.

### 3.3. Metabolism

Remdesivir is a prodrug which is metabolized through hydrolysis reaction to its triphosphate active form (GS-443902) [2]. The prodrug is metabolized by carboxylesterase 1 (CES1) (80%), cathepsin A (10%), and CYP3A (10%) to the extent indicated in parenthesis [15,18]. In vitro, CYP2C8 and CYP2D6 were also found to have some role in remdesivir metabolism [15]. GS-704277 is metabolized by the Histidine Triad Nucleotide-binding Protein 1 (HINT-1). Most of the metabolites (74%) are recovered in urine and about 18% are found in feces [15,18]. The nucleoside core metabolite (GS-441524) was the predominant remdesivir detected in urine with only 10% recovered as parent remdesivir prodrug [24]. Hepatic clearance of remdesivir is driven by hepatic blood flow rather than metabolic enzymes; this is believed to be true because of the high extraction ratio and shorter elimination half-life of remdesivir [21].

### 3.4. Elimination

The nucleoside core (GS-441524) of remdesivir is the most abundant derivatives identified in urine with remdesivir and other metabolites having moderate to low presence [25]. Biotransformation is the primary path of parent remdesivir and GS-704277 (alanine metabolite) excretion, whereas GS-441524 is eliminated by renal mechanisms such as glomerular filtration and active tubular secretion. Approximately 10% of the parent remdesivir is eliminated in urine as metabolite. In contrast, 49% of the dose is eliminated as GS-441524 through urine [18]. Remdesivir or alanine metabolites were not detected in the feces and <1% of the dose was excreted as the nucleoside core in the feces. GS-441524 has a half-life of 27 h compared to 1 h and 1.3 h for remdesivir and GS-704277, respectively [15]. The intracellular nucleoside triphosphate half-life is 14 h to 24 h, and the plasma half-life is about 1 h [21]. The nucleoside triphosphate remains the cellular compartment for a longer period due to the anionic charge on the molecule [21]. The half-life of the nucleoside triphosphate of remdesivir allows for the administration of once daily doses [25]. Since remdesivir is mainly excreted by the kidneys, it is not recommended to patients with renal impairment or patients undergoing renal replacement therapies such as dialysis or hemodialysis [15]. A patient with an eGFR ≥30 mL/min can take remdesivir and does not need a dosing adjustment, but once the eGFR is less than 30 mL/min, remdesivir is no longer recommended.

## 4. Pharmacokinetics of Remdesivir

Though originally remdesivir was studied for Ebola virus, the emergence of COVID-19 diverted the focus onto SARS-CoV-2 as remdesivir is an inhibitor of the SARS-CoV-2 RNA-dependent RNA polymerase enzyme [16]. Remdesivir is administered as a slow intravenous infusion, which allows it to reach to the therapeutic levels. Due to the extensive first-pass effect and consequent subtherapeutic levels, the oral administration of remdesivir is not desirable [15]. It also accumulates in muscle tissue, making intramuscular administration less favorable to reach therapeutic levels [15]. Thus, remdesivir PK profiles have been carried out with 200 mg/day of loading dose, followed by 100 mg/day of maintenance dose between days 2 and 5. For more severe patients that need mechanical ventilation and/or extracorporeal membrane oxygenation, the multiple-dose regimen of remdesivir is recommended for a total of 10 days [18].

The PK properties of the parent remdesivir, nucleoside core (GS-441524), and alanine metabolite (GS-704277) have been explored in different clinical studies in a limited way [15]. Tempestilli et al. [25] reported a PK study of the parent remdesivir and nucleoside core with two severely infected COVID-19 patients by administering a dosing regimen of a 200 mg/day loading dose, followed by 100 mg/day for the subsequent twelve days. Likewise, a study with healthy volunteers that received 200 mg/day of remdesivir, followed by 100 mg/day for next four days reported pharmacokinetic parameters [26]. In both the studies, remdesivir and GS-441524 reached to peak concentrations within 1 h [25,26]. The peak serum concentration achieved immediately after intravenous (IV) infusion was an average of 3027 ng/mL, which was followed by a quick drop in plasma concentrations after 1 h. The area under the curve (AUC) of the parent remdesivir in 24 h ranged from 2.9 µg·h/mL to 4.0 µg·h/mL [25]. GS-441524 exhibited different pharmacokinetic properties than remdesivir. The plasma concentrations measured 1 h and 4 h after infusion exhibited 214 ng/mL, 316 ng/mL, and 206 ng/mL, and 113 ng/mL, 184 ng/mL, and 92.6 ng/mL in patient 1 and patient 2, respectively. The AUC of the nucleoside core (GS-441524) at 24 h has been reported as being between 3.11 µg·h/mL to 6.13 µg·h/mL [25]. In comparison, the remdesivir Cmax on day 1 and day 5 was 5.44 µg/mL and 2.61 µg/mL, respectively, and for GS-441524, it was 0.15 µg/mL and 0.14 µg/mL, respectively. The AUC of parent remdesivir on day 1 and day 5 was 2.92 µg·h/mL and 1.56 µg·h/mL and for GS-441524 2.24 µg·h/mL and 2.23 µg·h/mL, respectively [26]. In a comprehensive dose escalation study with healthy volunteers, single or multiple dose regimens of remdesivir were studied for up to 14 days. Remdesivir (3 mg to 225 mg) was given as an IV infusion for 2 h and generated a linear kinetics profile [23]. The Cmax ranged from 57.5 ng/mL to 4420 ng/mL after a single dose of 3 mg to 225 mg remdesivir, each dose taking 2 h to reach to the peak plasma concentration. Similarly, the AUC ranged from 67.1 ng/mL to 5260 ng/mL. The clearance and volume of distribution ranged from 755 mL/min to 719 mL/min and 45.1 L to 66.5 L, respectively. Similar dose-dependent trends of Cmax and AUC were observed for GS-441524 and GS-704277 as well. Significantly higher (220–370-fold) active triphosphate cellular concentrations were observed after 75 mg or 150 mg single IV infusion compared to in vitro EC_50_ values observed in SARS-CoV-2 experimental models [23]. Interestingly, the parent remdesivir prodrug PK characteristics for 150 mg/day either for 7 days or 14 days were comparable. However, the GS-441524 and GS-704277 were somewhat different between day 1 and day 7 or day 14. The alanine metabolite increased approximately 1.9-fold following daily administration [23].

The current knowledge of remdesivir in special populations such as renal or hepatic impairment is very limited with discrete reports of remdesivir PK in COVID-19 patients with renal dysfunction. In silico simulation of remdesivir pharmacokinetics suggests that age, weight, liver, and renal function status can influence its disposition [27]. Since remdesivir and its active metabolite are excreted through urine [25], it necessitates the dose evaluation data on patients with renal impairment. The plasma concentration of remdesivir is increased in patients with chronic kidney disease [25,28,29]. The key PK parameters of parent remdesivir such as AUC_0-infinity_, Cmax, clearance, and volume distribution differ by 2.5-fold to 4-fold between healthy volunteers and renally impaired patients [30]. The FDA has outlined that remdesivir should not be given to patients with eGFR less than 30 mL/min [15,18]. However, two clinical trials of remdesivir observed acute kidney injury as one of the most prevalent adverse effects [28,29]. Remdesivir renal impairment could possibly be due to its solubility enhancer sulfobutylether β-cyclodextrin sodium (SBECD) in its formulations [15]. SBECD is found in various renally toxic drugs (e.g., IV formulation of voriconazole) and is eliminated renally with accumulation can occur at creatinine clearance (CrCl) <50 mL/min [31]. However, studies have also shown that regardless of accumulation in renally impaired patients, SBECD does not appear to cause severe renal toxicity [32,33]. It is still recommended to monitor renal function when administering remdesivir [15,18]. Interestingly, remdesivir can lower inflammatory response during high fat diet-related non-alcoholic fatty liver disease (NAFLD) in mice. The suppression of stimulator of interferon genes (STING), which is an endoplasmic reticulum membrane protein involved in innate immune response, has been identified as the central theme of remdesivir-mediated blockade of NAFLD progression [34]. This unfolds the plausibility that obese individuals with COVID-19 and liver dysfunction might experience dual benefits from remdesivir treatment, and eventually indirectly affect remdesivir disposition, therapy, and safety.

## 5. Potential Drug-Drug Interactions Involving Remdesivir

Enzyme-mediated bioactivation or inactivation of drugs can occur in most of the organs in mammals. However, the liver and small intestine are the main organs responsible for bulk of the drug metabolism in vivo [35]. As such, there are 57 isoforms of human CYP enzymes that are responsible for the biotransformation of endobiotic and xenobiotic substances including COVID-19 medications [36]. CYP3A4 is a critical enzyme that is responsible for about 70% of the drugs available in the clinic [37]. However, there are additional major CYP enzymes including CYP2C9, CYPC19, CYP2C8, CYP2D6, and CYP1A2 [35]. In addition to their ability to get inhibited or induced, the interindividual variability from genetic differences can have a differential effect on how they metabolize different drug substrates [38]. Though CYP enzymes are a major drug metabolizing enzyme superfamily, in recent years, additional significant enzymes such as esterases and aldehyde oxidases have been found to have roles in drug disposition [39].

Our analyses of COVID-19 treatment guidelines from three different agencies such as the Infectious Diseases Society of America (ISDA), the World Health Organization (WHO), and the National Institutes of Health (NIH) suggest that a host of drugs are prescribed in combination during different stages of COVID-19 [4,13]. The medications can be broadly classified into several groups including antivirals (e.g., remdesivir, lopinavir, ritonavir, umifenovir, favipiravir), antiprotozoal drugs (e.g., hydroxychloroquine, ivermectin), convalescent plasma, anti-bacterial agents (e.g., azithromycin), anti-inflammatory/immunosuppressants (e.g., corticosteroids, anakinra, interferon beta, monoclonal antibodies such as tocilizumab, cocktail to neutralize inflammatory proteins), and supportive agents (e.g., propofol, famotidine, benzodiazepine) [4,12,13,40]. Table 1 depicts the CYP substrate, inhibitor, or inducer profile of representative drugs used in COVID-19 treatment regimens. In addition, retrospective studies with COVID-19 patients have shown that individuals with preexisting conditions or comorbidities (e.g., hypertension, diabetes, hyperlipidemia) are more susceptible to the virus [41,42]. According to the Centers for Disease Control and Prevention (CDC) the most serious risk factors to severe COVID-19 infections are adults ≥ 65 years old, health care workers such as nurses in long-term care facilities, patients who are immunocompromised, and patients with pulmonary, cardiovascular, and endocrine diseases [9]. This indicates that COVID-19 patients will be on additional medications (e.g., antihypertensives such as amlodipine, nifedipine, irbesartan, losartan, verapamil, and diltiazem; antidiabetic agents such as glimepiride, glipizide, glyburide; cholesterol lowering drugs such as statins) to manage their comorbidities [41,42]. Since significant polypharmacy is employed to manage highly infectious and virulent SARS-CoV-2, ADME-related drug interactions between the COVID-19 drugs are highly likely in patients.

Drug-drug or drug-herb interactions, which will be generically termed as DDI in this discussion, can have at least two major perspectives of “perpetrator” and “victim”. Thus, it is important to understand how a particular drug/natural product is metabolized by CYP or non-CYP enzymes and how other drugs/natural products can affect the metabolism of the drug in question (“victim”) through the induction or inhibition of metabolizing enzymes. Likewise, the knowledge of induction and inhibition profile of the drug in question is important to predict and characterize its effects on the metabolism of other co-administered agents (“perpetrator”). In this light, the remdesivir has an interesting ADME profile that makes it a good candidate for DDI as “victim” as well as “perpetrator” (Table 2).

**Figure 2:**
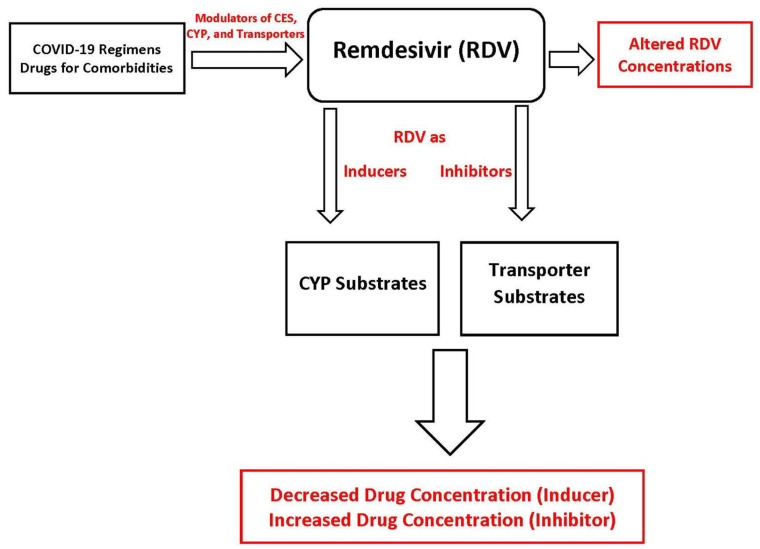
Potential drug-drug interactions involving remdesivir, either as “victim” or “perpetrator”. CYP, cytochrome P450, CES, carboxylesterase.

The transporters, CYP enzymes, and non-CYP enzymes (e.g., CES1) can play key roles in the DDI landscape of remdesivir [18]. The DDIs of remdesivir should be considered from two critical perspective of disease pathophysiology and management, namely, agents that are used in the management of COVID-19 and its symptoms, and agents that are used by the patients in managing the comorbidities (Figure 2). Screening of available dose regimens suggests that dexamethasone, hydroxychloroquine, and antibiotics used to manage symptoms can cause DDIs [24,42]. Likewise, it is well known that individuals with certain conditions such as hypertension and diabetes are more susceptible to SARS-CoV-2 infection [41,42]. This suggests that antihypertensive or antidiabetic agents used in COVID-19 patients to management their preexisting conditions have the potential to affect the disposition of remdesivir and vice versa [41,42]. Indeed, our recent simulation work predicted that COVID-19 regimen related other drugs (e.g., ritonavir) and agents for comorbidities (e.g., voriconazole, diltiazem) have the potential to interact with remdesivir [27]. To understand the potential drug-drug interactions of remdesivir, we must identify drugs that may be given before, with, and immediately after remdesivir administration. In an ideal scenario all drugs should be assessed for drug-drug interactions with remdesivir. However, the focus of the clinical analyses should be on drugs used to treat COVID-19 and drugs employed to treat preexisting comorbidities.

### Remdesivir as “Victim”

Though detail in vitro or in vivo metabolism study of remdesivir is not available at this time, it appears that remdesivir is a substrate of CES1 (80%), Cathepsin A (10%), and CYP3A (10%) with minor roles of CYP2C8 and CYP2D6 [18]. The substrate profile of remdesivir with P-gp and OATP1B1 also offers reasonable possibility of mechanisms through which its plasma concentrations can be affected [18]. In addition, there is very limited to no information available about the metabolism of multiple remdesivir metabolites that are produced in the bioactivation cascade and eventually responsible for the antiviral actions of remdesivir.

Remdesivir disposition appears to be affected by P-gp modulators. Indeed, a COVID-19 patient case study by Leegwater et al. [54] reported that remdesivir can induce liver toxicity when it is co-administered with amiodarone and administered nine days after chloroquine dosing. It has been postulated that plasma concentrations of remdesivir increased due to the inhibition of P-gp by amiodarone [54]. Amiodarone is commonly used to treat abnormal heart rates and can inhibit P-gp, CYP3A4, and other enzymes as well [52]. P-gp acts as a transmembrane efflux pump where it can remove remdesivir out of the hepatic cell; however, the inhibition of P-gp can block the hepatic clearance of remdesivir, thus trapping the drug inside the hepatocytes, leading to hepatotoxic concentrations. Following the discontinuation of remdesivir, the liver enzymes of the patient returned to within normal limits [54]. This suggests that P-gp inducers (e.g., phenytoin, rifampin, St. John’s wort) and inhibitors (e.g., amiodarone, lopinavir, ritonavir) have the potential to affect the movement of “victim” remdesivir in the hepatic and other tissue compartments [52].

It is critical to realize that so far CES1 has been identified as the major enzyme in remdesivir metabolism. CES1 is one of the most abundant drug metabolizing enzymes and its levels are >5 fold higher than the most abundant CYP enzymes [60]. Thus, non-CYP interaction could play a major role in the disposition of remdesivir. For example, dexamethasone, which has been one of the mainstays of COVID-19 regimens, is an inducer of CES1 [61]. Likewise, drugs used to manage COVID-19 comorbidities such as cardiovascular diseases (e.g., telmisartan, carvedilol, diltiazem, nitrendipine, procainamide, quinidine) and hyperlipidemia (e.g., simvastatin, fenofibrate) can inhibit CES1 [62,63] and block the conversion of remdesivir to its intermediate and active metabolites. In addition, several commonly used diet and natural health supplements taken by the COVID-19 patients can influence its outcome. Grapefruit juice, ginsenosides, cannabinoids, and resveratrol have been reported as some of the notable human CES1 inhibitors [60]. To further complicate the matter, CES1 is known to have genetic polymorphism [64], which suggests that every COVID-19 patient on remdesivir will not have the same outcome in terms of a CES1-mediated metabolism. Considering the abundance of CES1 and ubiquity of its inducers and inhibitors in the COVID-19 treatment regimen or in drugs for comorbidities, it is highly likely that remdesivir is affected by non-CYP drugs interactions.

In terms of CYP-related interactions with remdesivir, there are ample opportunities of modulating remdesivir metabolism due to the CYP inducers and inhibitors administered in COVID-19 patients [37]. Though CYP3A4 is reported to have about 10% contribution in the metabolism of remdesivir [65], depending on the relative status of non-CYP metabolizing enzyme activities, CYP3A4 may contribute to a higher extent. This is also applicable for other CYP enzymes, which apparently have lower involvement in the basal conditions but can contribute more when CYP3A4 activities are suppressed or inhibited.

## 6. Future Directions of Remdesivir ADME and DDI Research

Remdesivir has shown much potential to combat SARS-CoV-2 infection, however, this treatment option currently lacks patient-friendly dosage forms, sufficient toxicity, and drug interaction data. In addition to the currently available parental dosage forms, a major opportunity for improvement is the development of inhaled dosage forms for localized absorption and distribution. The current dose regimen of remdesivir may yield a subpar concentration in inhibiting COVID-19 in the lungs, which is the primary site of attack by SARS-CoV-2 [21]. The inhaled administration would allow the medication to be absorbed into the lungs in therapeutic concentrations to effectively treat and kill SARS-CoV-2. An inhaled administration can also be beneficial in decreasing systemic toxicities by giving a smaller dose more locally into the infected area. Inhaled administration will likely be for less serious COVID-19 patients since it would be for local treatment [21].

The potential drug-drug interactions could complicate the treatment outcomes of COVID-19. The case report outlined by Leegwater et al. [54] is a classic example that drugs used with remdesivir should be assessed for possible ADME interactions before, during, and after treatment for COVID-19. As the ADME, PK, and pharmacodynamics details of remdesivir become available, healthcare professionals will be in a better position to prevent remdesivir-related DDIs in COVID-19 patients. In addition, remdesivir has shown the potential to cause liver toxicity through hepatocellular injury, though the exact mechanism by which the hepatocellular injury occurs is currently unknown [66]. The increase in liver enzymes is a concern due to the extensive number of hepatotoxic medications that a COVID-19 patient must take to manage a wide range of symptoms and complications. Additional research is needed on remdesivir’s ADME interactions with COVID-19 drug regimens and drugs used to treat preexisting conditions for better treatment outcomes.

Overall, remdesivir is an adenosine nucleotide analog prodrug which is metabolically activated to a nucleoside triphosphate metabolite (GS-443902). Transporters, CYP enzymes, and CES1 are involved in remdesivir disposition. The intravenous administration of remdesivir affords patients to achieve therapeutically meaningful plasma concentrations. Special populations with hepatic and renal dysfunction have demonstrated indications of differential pharmacokinetics and have the potential for toxicity from remdesivir. Finally, due to the contribution of diverse ADME proteins in remdesivir disposition, the drug-drug interaction potential involving remdesivir is high. This is even more critical because of the ability of remdesivir to act as “victim” or “perpetrator” in drug interaction scenarios. There is an urgent need for mechanistic experimental work on drug-drug interactions using rodent models, cell culture models, and human tissues. Nonetheless, the current knowledge of remdesivir ADME and PK shall allow the healthcare professionals to identify DDI and toxicity situations in a limited manner.

## Figures and Tables

**Table 1:** COVID-19 drug regimens and their classification as CYP inhibitors and inducers.

Drug	Substrate	CYP Inhibitor	CYP Inducer
Remdesivir [15,18,24]	CES, CYP3A4, CYP2C8, CYP2D6, P-gp, OATP1B1	CYP1A2, CYP2C9, CYP2C19, CYP2D6, CYP3A4	CYP1A2, CYP2B6
Dexamethasone [43]	CYP3A4, P-gp	CYP3A4	CYP3A4
Chloroquine/Hydroxychloroquin [44]	CYP2C8, CYP3A4/5, CYP2D6	CYP2D6	None reported
Azithromycin [45]	CYP3A4, P-gp	None reported	None reported
Lopinavir/Ritonavir [46]	CYP1A2, CYP2B6, CYP2D6, CYP3A4, P-gp	CYP2D6, CYP3A4	CYP1A2, CYP2B6, CYP2C19, CYP2C9
Ivermectin [47,48]	CYP3A4, P-gp	CYP2C9, CYP2D6, CYP2C19, CYP3A4	CYP1A, 2B and 3A
Anakinra [49]	None reported	None reported	None reported
Interferon beta [50,51]	None reported	CYP1A2	None reported

**Table 2:** Potential drug-drug interactions of remdesivir with COVID-19 treatment regimens and with drugs for comorbidities.

Drug	Process Modified	Potential Outcome
Drugs Affecting Remdesivir ADME		
Amiodarone [52,53,54]	Inhibition of P-gp and CYP	Increased remdesivir concentration
Chloroquine [55,56]	Inhibition of P-gp	Increased remdesivir concentration
Fluoxetine, Grapefruit juice [57]	Inhibition of CES	Increased remdesivir concentration
Dexamethasone, Rifampin [58]	Induction of CES	Decreased remdesivir concentration
Phenytoin, St. John’s Wort [52,59]	Induction of CYP3A4	Decreased remdesivir concentration
Lopinavir-ritonavir [46]	Inhibition of CYP3A4	Increased remdesivir concentration
Drugs Affected by Remdesivir		
Theophylline [15,18,24]	Induction of CYP1A2	Decreased theophylline concentration
Olmesartan [15,18,24]	Inhibition of OATP 1B1/1B3 transporters	Increased olmesartan concentration
Warfarin [15,18,24]	Inhibition of CYP2C9	Increased warfarin concentration
Irinotecan [15,18,24]	Inhibition of OATP 1B1	Increased irinotecan concentration
Atorvastatin [15,18,24]	Inhibition of CYP3A4	Increased atorvastatin concentration
Metformin [15,18,24]	Inhibition of MATE1	Increased metformin concentration

## Data Availability

Data sharing not applicable.
